# Performance and Behavioural Responses of Group Housed Dairy Calves to Two Different Weaning Methods

**DOI:** 10.3390/ani9110895

**Published:** 2019-11-01

**Authors:** Gillian Scoley, Alan Gordon, Steven Morrison

**Affiliations:** 1 Agri-Food and Biosciences Institute, Hillsborough, BT26 6DR, UK; steven.morrison@afbini.gov.uk; 2 Institute for Global Food Security, School of Biological Sciences, Queen’s University Belfast, Belfast BT7 1NN, UK; 3 Agri-Food and Biosciences Institute, Newforge Lane, Belfast BT9 5PX, UK; alan.gordon@afbini.gov.uk

**Keywords:** dairy calf, feeding behaviour, milk replacer delivery, monitoring technologies, weaning method

## Abstract

**Simple Summary:**

Removal of the milk feed, more commonly known as weaning, is considered as a major stressor in the early life of a calf, which, if handled improperly, can have a negative impact on both calf performance and well-being. Traditional methods of assessing the impact of weaning have involved behavioural observations and blood sampling, which can be impractical for farmers and invasive for animals involved. Recently, developments in technology have increased the availability of on-farm non-invasive devices which allow automatic and remote collection of behavioural and physiological data linked to animal health and well-being. This study aimed to use conventional measures of animal performance in combination with non-invasive monitoring technologies to examine the impact of two different weaning methods in group housed calves. Results indicated that both gradual and abrupt methods of weaning described in this study can be used without significant impact to calf live weight at 63 days of age. However, data from the monitoring technologies indicated that weaning method can have an impact on calf behaviour and use of automatic milk and concentrate feeders, providing information on overall calf well-being. The results of this study indicate how monitoring technologies can be incorporated on-farm and how the information can be used to highlight potential areas of increased calf frustration within common calf management practices.

**Abstract:**

The weaning of dairy calves is a significant stressor which can impact on calf performance and welfare. However, many traditional methods of assessing the effects of stressors can be invasive and impractical for farmers. This study aimed to use a combination of non-invasive monitoring technologies alongside traditional measures of calf performance to examine the impact of two contrasting weaning methods commonly used on dairy farms in the United Kingdom. Ninety group-housed Holstein Friesian calves were allocated to one of two weaning methods: (i) gradual weaning (GW) with volume of milk replacer (MR) stepping down from 36 days of age and complete withdrawal of MR at 57 days of age and (ii) abrupt weaning (AW) with consistent daily volume of milk replacer and complete withdrawal of MR at 50 days of age. Feeding regimes were such that calves from both treatments were offered the same total amount of milk powder. Gradually weaned calves displayed increased solid feed intake at an earlier age when compared with AW calves. Feed conversion efficiency (FCE) was reduced in gradually weaned calves between days 36 and 49. However, there was no difference in live weight (LWT) or average daily gain (ADG) throughout this period. Abrupt weaning at 50 days of age resulted in decreased ADG and FCE between days 50 and 56. However, there were no treatment differences in ADG between days 57 and 62. Live weight tended to be increased by 2.2 kg in GW calves when compared with AW calves at the end of experiment on day 63. Frequency of unrewarded visits to the milk feeder throughout the pre-wean period was consistently increased in GW calves. Daily lying time was reduced in AW compared with GW calves in the days following abrupt weaning (days 50–55). However, these differences did not persist between days 57 and 62. Heart rate variability (HRV) tended to be decreased in GW compared with AW calves in the period following complete withdrawal of milk replacer. Findings from the current study suggest that calves offered the same total amount of milk powder can be weaned either gradually from 36 days of age or abruptly at 50 days of age without significant impact to live weight at 63 days of age. However, both behavioural and physiological data collected using the methods described could suggest that gradual weaning of calves from 36 days of age results in an increase in underlying frustration. This study highlights the potential of using a combination of non-invasive monitoring technologies in assessing calf response to common management practices.

## 1. Introduction

Weaning is a multifaceted stressor incorporating nutritional, environmental and physiological elements and thus is one of the most challenging periods for both producers and calves (e.g., [1]). Improper handling of stressors in early calfhood can impact negatively upon future health, welfare and productivity [2]. Abrupt weaning of calves, whereby a previously unchanged milk allowance is removed suddenly over 24 h, has been associated with decreased solid feed consumption in the pre-wean period and a decrease or stall in growth rate in the period following the withdrawal of milk [3]. Practices such as step-down or gradual weaning through reduction in volume of milk over a number of days or reduction in number of milk meals have been suggested as a means of reducing the impact of weaning stress. Sweeney et al. [4] examined the impact of weaning duration on concentrate intake and performance of calves offered high levels of milk. Concentrate intake was increased in calves subject to 10 or 22 day weaning durations when compared to those weaned either abruptly or over 4 days. However, the increased intake was not sufficient to compensate for the reduced intake of milk, leading to lower digestible energy intakes and low live weight gains in the calves weaned over 22 days. Another method of gradual weaning is to increase dilution of milk replacer. This method was used by Overvest et al. [5] in pair housed calves as it was considered that if a volume reduction method had been implemented there could have been greater variation in milk intake between calves. These gradual weaning methods have been shown to encourage solid feed intake at an earlier age which is thought to help prepare calves for a solid feed diet and thus lessen the impact of milk removal (e.g., [3,4]). However, these studies also highlight the importance of considering calf age, concentrate intake and milk feeding system when choosing a weaning method so as to ensure calves achieve performance targets.

Calf reaction to stressors such as weaning has traditionally been assessed using behavioural and aural observations (e.g., [6]). Recently, however, it has been suggested that the use of combined remote sensing technologies could allow measurement of several behavioural and physiological responses, which, when compared with measuring only a single aspect, could provide a more accurate aggregate indicator of animal well-being [7]. The use of automatic milk feeders and pedometers has provided increased opportunities for behaviour monitoring in a remote, non-invasive manner [8]. With regard to long-term measurements, automated feeders allow precise measurement of individual feed intake (e.g., [9]) and the recording of behavioural traits, deviations in which can be indicative of increased stress as a result of feeding regime [10]. Measurement of calf activity using pedometers or accelerometers has previously been linked with hunger, with De Paula Vieira et al. [11] finding that standing time was increased by 1 hr in limit fed calves when compared with those fed ad libitum. With regard to underlying physiological response to stressors, recent research has highlighted heart rate variability (HRV) as a viable, non-invasive parameter for measuring response to environmental, physiological and psychological stress load [12,13,14].

With an increasing range of on-farm sensing technologies available, the aim of this study was to determine the effect of gradual versus abrupt weaning on group housed calves using non-invasive, automated behavioural and physiological parameters. A further objective was to assess the impact of weaning method on calf performance parameters.

## 2. Materials and Methods

This study was conducted at the Agri-Food and Bioscience Institute (AFBI) research farm in Hillsborough (54°27′ N; 06°04′ W). All procedures and treatments within this study approved by the Agri-Food and Biosciences Institute Animal Welfare and Ethical Review Body and conducted under a license from the Department of Health, Social Services & Public Safety for Northern Ireland in accordance with the Animals (Scientific Procedures) Act 1986.

### 2.1. Animals

Ninety Holstein Friesian calves (39 females and 51 males) born between 19th September and 10th December were allocated to the study following manual weighing at ≤12 h of age (41 ± 4.4 kg). All calves received 3 L colostrum via oesophageal feeding tube or teated bottle before transfer to the calf-rearing unit. Single point blood samples were taken via jugular venipuncture using 10 mL clot activated serum separation vacutainer tubes (BD, Plymouth, UK) at 24–36 h old. Samples were analysed using the zinc sulphate turbidity (ZST) technique as described by McEwan et al. [15] to determine effectiveness of passive transfer. Calf immune status was assessed by analysis of immunoglobulin G (IgG) levels using the BioX ELISA test (Bio-X Diagnostics, Jemelle, Belgium) as described by Dunn et al. [16]. All calves allocated to the study were determined to have a ZST value of >20 units, which is indicative of successful passive transfer of immunity [15] and IgG level was comparable across treatments.

Calves were vaccinated with Bovilis Bovipast RSP (MSD Animal Health, Milton Keynes, UK) via subcutaneous administration at 2 weeks of age with a second dose given 4 weeks later at which point calves were also vaccinated with Bovilis IBR Marker Live (MSD Animal Health, Milton Keynes, UK) via intranasal administration.

### 2.2. Treatments and Experimental Design

On entering the rearing accommodation at ≤12 h of age calves were individually penned (1.8 m length × 1 m width) and allocated to one of two weaning treatments: (i) gradual weaning (GW) with volume of milk replacer (MR) stepping down from 36 days of age and complete withdrawal of MR at 57 days of age and (ii) abrupt weaning (AW) with consistent daily volume of milk replacer and complete withdrawal of MR at 50 days of age (Table 1). Milk replacer was offered at a rate of 150 g/L and weaning protocols ensured that all calves were offered the same total quantity of milk powder.

### 2.3. Housing and Diet

Calves were fed 2 L colostrum twice daily via teated bucket on days 1–3. At 4 days of age, calves were provided with a 2 L feed consisting of a mix of half colostrum/half milk replacer in the morning and afternoon. At 5 days of age calves were introduced to one of six straw bedded pens (6 m length × 6 m width) of no more than 15 calves and all liquid feeds were of a whey-based milk replacer (Volac International Ltd., Hertfordshire, UK). One automatic milk feeder serviced 2 group pens with each pen having 1 milk feeding station equipped with 1 teat from which calves were offered milk replacer according to weaning regime. Each group pen consisted of a mix of calves from both treatments; however, this was balanced across each automatic milk feeder so that 15 calves from each treatment were serviced by each of the three automatic feeders. Calves had ad libitum access to concentrate starter feed via 1 automatic concentrate feeder per group pen equipped with 1 feeding station, ad libitum straw from racks and free access to fresh water for the duration of the experimental period. Calves remained in the same group pen until the end of experiment at 63 days of age and no calves were excluded from the study.

### 2.4. Data Collection

#### 2.4.1. Feed Nutrient Composition

Samples of milk replacer, starter concentrate ration, and fresh straw bedding were collected on a daily basis and bulked for each 2 week experiment period. The samples were analysed using the methods as described by Dunn et al. [17]. Chemical composition of feedstuffs is presented in Table 2.

#### 2.4.2. Live Weight

Live weight, as measured using a calibrated manually operated weighbridge (Tru-Test Eziweigh 5, Auckland, New Zealand) was recorded at birth and at days 50, 57 and 63 of age in addition to every Monday morning between 10:00 and 12:00 for the duration of the study. Calf live weight was also recorded automatically on a daily basis via the half body weight scales linked to the feed station of the automatic milk feeder.

#### 2.4.3. Feed Intake and Feeding Behaviour

Individual daily milk and concentrate intake was recorded via the automatic feeder between 5 and 62 days of age. Automatic feeders recorded individual calf feeding behaviour information, including the timing and duration of visits. In addition, the automatic milk feeder recorded individual calf drinking speed and number of rewarded (MR received) and unrewarded (no MR received) visits.

#### 2.4.4. Calf Health

Faecal consistency was qualitatively scored on a daily basis for each individual calf during morning feeding time using the scale of 1 = normal consistency, 2 = slightly liquid consistency, 3 = moderately liquid and 4 = primarily liquid consistency. A calf was recorded as having scour when the score was greater than two [18]. Respiratory disease scoring, with the exception of rectal temperature, was carried out on a daily basis using the University of Wisconsin–Madison method [19]. Both faecal and respiratory scoring were carried out by a trained technician. Cases of calf ill health were assessed on an individual basis and treatment was administered according to predefined protocols as provided by a veterinarian.

#### 2.4.5. Calf Activity

IceRobotics^®^ IceQube^®^ automatic activity sensors (IceRobotics Ltd., Edinburgh, Scotland, UK) were fitted to 5 calves per experimental pen (15 calves per treatment) between the ages of 42 to 62 days to allow data capture over the pre- and post-weaning periods for both treatments. Selected calves were balanced across treatments for sex and birth weight. The automatically generated standing and lying bout duration data were downloaded from the sensors with any lying bout durations ≤8 s removed prior to analysis [20].

#### 2.4.6. Heart Rate Monitors

Polar Equine RS800CX Science (Polar Electro UK Ltd., Heathcote Way, Warwick, UK) heart monitors were fitted to the same calves fitted with automatic activity sensors. Heart monitors were fitted to calves on days −2, −1, 0, +1 and +2 where day 0 = day of complete withdrawal of milk replacer. After ensuring that all calves within the experimental pen had consumed the morning milk replacer allocation, selected calves were moved to the corner of the group pen and penned in. Sampling was conducted between 09:00 and 12:00 and calves remained in the penned area for the duration of sampling. Monitors were fitted and remained on calves for approximately 1 h to allow data capture during the period when calves were undisturbed and observed to be lying down. Electrode gel (Spectra 360 Electrode Gel, Parker Laboratories Inc., Fairfield, NJ, USA) was applied to ensure contact between the calf and monitor [12]. Data sets of 5–10 min duration when calves were determined to be at rest [21] were selected for each calf for each day and processed using both the Artiifact and Polar Pro Trainer software [22]. Preliminary error correction was conducted using Polar software [12], with any data sets requiring over 5% error correction discarded. Further data processing was then conducted with Artiifact software, with the resultant root mean squares of successive differences (RMSSD) in inter heart beat intervals (IBI) used in the analysis [13].

### 2.5. Statistical Analysis

All data were analysed using GenStat^®^ (version 16.2, VSN International Ltd., Hemel Hempstead, UK). Models were selected based on normality of each data set. All statistical models included birth weight as a covariate and pen as a random term unless otherwise stated. Statistical significance was determined at *p* < 0.05 and a tendency was determined at *p* < 0.1. Where data was significant it was subjected to post hoc testing using Fisher’s protected least significant difference (PLSD) test. Feed intake and automatic feeder behavior data were classified into 4 time periods; day 5–35 (Period 1; baseline), day 36–49 (Period 2; reduction of MR provision in GW calves), day 50–56 (Period 3; post-weaning in AW calves and reduction of MR provision in GW calves) and day 57–62 (Period 4; post-weaning in GW calves) and analysed individually by period. A covariate derived by averaging data from the 5 days immediately prior to the commencement of the next period was included in all analysis pertaining to periods 2–4.

#### 2.5.1. Calf Performance and Feeding Behavior

Calf live weight, daily concentrate intake, daily milk replacer (MR) intake and time spent at the automatic feeders were fitted to a repeated measures residual maximum likelihood estimation (REML) model with effects of sex, time, treatment and the interaction of treatment × time included. Number of visits to the automatic feeders were fitted to a generalized linear mixed model (GLMM) using a C.F. Schall method and Poisson distribution with effects of sex, age, treatment and the interaction of treatment x time included. Live weight gain and total concentrate, MR and dry matter intake were analysed using a linear mixed-effects model with fixed effects of sex and treatment.

#### 2.5.2. Calf Health

Average faecal and respiratory score for each individual calf across the experimental period were analysed using a mixed-effects model with fixed effects of sex and treatment. Number of scour and pneumonia episodes per calf were each fitted to a GLMM using a C.F Schall method and Poisson distribution with effects of sex and treatment included.

#### 2.5.3. Physiological and Behavioural Measures

Within lying behaviour parameters, pre-weaning data were apportioned as the 5 days prior to complete withdrawal of milk for each weaning plan and post-weaning data were those pertaining to the day of complete milk withdrawal and the following 5 days. The pre- and post-weaning period data were then analysed individually. Heart rate data were split into two post-weaning periods, the day of weaning and 2 days following complete withdrawal of milk replacer for each weaning method. Each group was analysed individually for each post-weaning period using the aforementioned repeated measures model. A covariate derived by averaging the data from the two days prior to complete milk withdrawal was included in the analysis.

## 3. Results

### 3.1. Feed Intake and Calf Performance

Total volume of milk replacer consumed did not differ between treatments (Table 3; *p* = 0.685). As expected, GW calves consumed almost twice as much starter concentrate/day as AW calves during days 36–49 (Table 3; *p* < 0.001)) and AW calves consumed approximately 400 g DM/day more than GW calves during days 50–56 (Table 3; *p* < 0.001). No effect of treatment was observed on either daily or total concentrate DM intake during days 57–62 with calves from both treatments consuming greater than 2 kg DM starter concentrate/day (Table 3; *p* = 0.715). Total dry matter intake was greater in GW than AW calves between days 5 and 56 (Table 3; *p* < 0.001) and days 5 and 62 (Table 3; *p* = 0.001). Daily concentrate intake is presented in Supplementary Figure S1.

There was no effect of weaning plan on calf live weight as measured by manual weighbridge at days 50, 57 or 63 (Table 4). Daily live weight of calves is presented in Supplementary Figure S2. When assessed between days 7 and 62, GW calves had a slightly increased ADG when compared to AW calves, but this wasn’t statistically significant (Table 4; *p* = 0.087). There was an effect of weaning plan on ADG between days 50 and 56 with GW calves gaining 0.5 kg/day more than AW calves (Table 4; *p* < 0.001). Average daily gain was 1.0 vs. 0.86 kg/day respectively for AW and GW calves between days 57 and 62, but this was not significant (Table 4; *p* = 0.150).

When analysed between days 7 and 56, there were no treatment differences in feed conversion efficiency (feed conversion efficiency = kg of BW gain/kg of total DMI) (Table 4; *p* = 0.565). However, increased feed conversion efficiency (FCE) was observed in AW calves between days 36 and 49 when compared with GW calves (Table 4; *p* < 0.001). Between days 50 and 56, FCE was lower in AW calves compared to GW calves (Table 4; *p* = 0.049) however, the reverse was true between days 57 and 62 with increased FCE observed in AW calves (Table 4; *p* = 0.031).

### 3.2. Calf Health

There was no effect of weaning method on faecal score throughout the experimental period, with an average score of 1.1 across treatments (*p* = 0.943). There was no effect of treatment on number of scour episodes (*p* = 0.687), with 48.9% of calves from each treatment determined as experiencing an episode of scour (i.e., a score >2). Nor were there any differences in proportion of calves experiencing episodes of pneumonia (*p* = 0.407), with values of 37.8% and 42.2% for GW and AW calves, respectively.

### 3.3. Automatic Feeder Behaviour

#### 3.3.1. Drinking Speed and Drinking Time

Drinking speed of GW calves tended to be higher (*p* = 0.078) compared with AW calves in the latter stages of Period 2 (day 36–49). As expected, time spent drinking milk declined from 5.9 min/day at the start of Period 2 (day 36) to 3.2 min/day at the end of Period 2 (day 49; *p* < 0.001) in GW calves. There was no difference in AW calves with values of 5.8 and 5.5 min/day between the start (day 36) and end (day 49) of Period 2, for day 36 and day 49, respectively. In the same period, duration of time in the milk feeder without feeding was 18.2 and 14.6 min/day for GW and AW calves respectively (*p* < 0.001).

#### 3.3.2. Milk and Concentrate Feeder Visits

**Figure 1:**
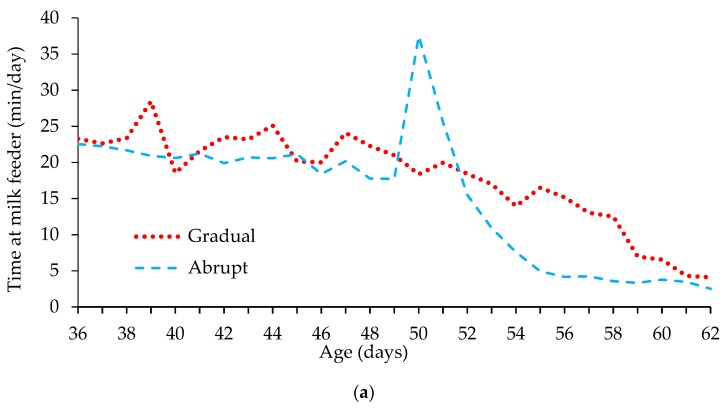

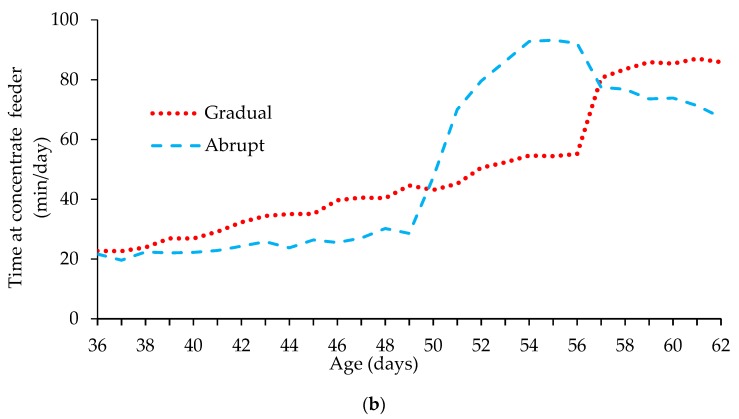
Time spent occupying the milk (**a**) and concentrate (**b**) feeders (min/day) between 36 and 62 days of age in calves weaned either gradually between 36 and 57 days of age or abruptly at 50 days of age.

Frequency of milk and concentrate feeder visit were as expected, and generally reflected the availability of MR due to the imposed weaning treatments within the study. As anticipated, no treatment differences were observed in feeder visit parameters during Period 1 (*p* > 0.05). During Period 2 (day 36–49) the total number of visits to the milk feeder was higher in GW calves when compared with AW calves (*p* < 0.001). Although visits were of a shorter duration (*p* < 0.001), the higher number of visits resulted in GW calves occupying the milk feeder for over 2 min/calf/day more than AW calves (Figure 1a; *p* < 0.001). Weaning of AW calves at day 50 resulted in an initial steep increase in number of unrewarded visits to the milk feeder (*p* < 0.001), however, this abated by day 52, with number of unrewarded milk feeder visits comparable between weaning treatments. The higher number of visits by AW calves also led to a greater time spent occupying the milk feeder in comparison with GW calves (Figure 1a; *p* < 0.001), however, this difference again only existed until day 52. Average visit duration was 30 s less or more in AW calves than GW calves between days 50 and 56 (*p* < 0.001). Gradually weaned calves displayed a greater number of unrewarded visits to the milk feeder following complete withdrawal of milk during Period 4 (day 57–62) when compared to AW calves (*p* < 0.001). Average visit duration was also lower in GW calves during this time (*p* = 0.022). GW calves spent 8 min per day more in the concentrate feeder than AW calves (*p* < 0.001) in Period 2 (day 36–49). During Period 3 (day 50–56) AW calves spent 80 min/day at the concentrate feeder whereas GW calves spent 51 min/day (Figure 1b; *p* < 0.001). During Period 4 (day 57–62) GW calves spent approximately 11 min per day more at the concentrate feeder than AW calves (*p* < 0.001). Diurnal patterns of milk and concentrate feeder occupation are presented in Supplementary Figure S3.

### 3.4. Lying Behaviour

Treatment had no effect on total daily lying time, number of lying episodes or lying episode duration in the five days prior to abrupt weaning (day 45–49). Abruptly weaned calves displayed a reduced number of lying episodes (Table 5; *p* = 0.004), and a decreased average daily lying time (Table 5; *p* < 0.001) in the period following withdrawal of milk at 50 days of age when compared with gradually weaned calves. There was no effect of treatment on number of lying episodes, episode duration and average total daily lying time in the period immediately following completion of gradual weaning.

### 3.5. Heart Rate Variability

There were no differences between treatment groups in RMSSD values on the day of or in the 2 days following complete weaning of AW calves (GW 32.3 vs. AW 41.0; *p* = 0.101), nor on the day of and 2 days following complete weaning of GW calves (GW 36.8 vs. AW 47.4; *p* = 0.067). However, when the abrupt and gradual post-weaning periods were directly compared, i.e., day 50–52 vs. day 57–60, RMSSD was lower in GW calves than AW calves (33.3 vs. 49.9; *p* = 0.002).

## 4. Discussion

### 4.1. Calf Performance and Efficiency

Juvenile animals primarily use available nutrients for maintenance of bodily functions with only excess nutrients directed towards growth [23]. Calves fed restricted levels of milk are often unable to meet daily energy requirement despite consuming increased amounts of solid feed when compared with calves with increased milk allowance (e.g., [3,23]), this likely the reason for reduced FCE and ADG observed in GW calves during day 36–49. The large reduction in available energy in AW calves as a result of MR withdrawal severely reduced ADG and FCE in the week following weaning when compared with GW calves. This was likely due to the fact that GW calves were experiencing less of a reduction in available energy than AW calves as they were established on over 1 kg/day solid feed in the days prior to weaning and milk replacer was reduced by only 300 g over 24 h as opposed to the 900 g reduction in AW calves. The age and duration of the gradual weaning program used in the present study is fairly typical of occurrences in commercial practice and used in recent research in calves fed increased levels of MR (e.g., [4,24]). Ideally, weaning programs should maximise milk intake in the first weeks of life whilst encouraging early consumption of solid concentrate feed. Further research to find optimum weaning protocols for producers using different management regimes are essential to help improve calf performance, health and welfare.

Previous research has suggested that gradual weaning can facilitate smoother transition to a solid feed diet due to increased solid feed intake in the pre-wean period [25]. However, despite the reduction in growth and feed efficiency experienced by AW calves in the week immediately following weaning, results of the present study showed that calves were able to recover quickly, with Period 3 (day 50–56) FCE and ADG doubling during Period 4 (day 57–62). The rapid increase in solid feed intake and swift recovery in performance observed in abruptly weaned calves in the present study suggests that, in accordance with findings from Steele et al. [24], calves can quickly adapt to a new feed source.

### 4.2. Automated Feeders

Nielsen [26] highlighted the value of automatic feed intake recording systems which can help provide long-term information regarding development of feeding behaviour, categorisation of feeding patterns which deviate from normal baseline behaviour. Within the present study, experimental pens consisted of calves from both treatments. It could be considered that this may have affected milk feeder behaviour, as calves within the same pen were being weaned at different times. This setup was chosen to reflect the dynamic nature of calf pens on commercial farms. The feeding behaviour observed in the present study was largely expected, and reflective of the imposed feeding treatments, but it does provide relevant information which could help inform best practice regarding age at commencement of gradual weaning. Calves fed according to a gradual weaning protocol from 36–49 days of age visited the milk feeder more frequently, experienced more unrewarded visits and spent an increased proportion of time in the feeder not consuming milk than calves on an unchanged milk allowance prior to weaning. An increase in unrewarded visits to the milk feeder has previously been observed in limit fed calves when compared with calves fed greater quantities of milk (e.g., [11]) and, as such, was suggested as an indicator of hunger. In a natural setting, calves would suckle between four and 10 times per day [27], so it could be considered that visits above that level are indicative of increased hunger and frustration, thus reduced calf welfare. The aforementioned behaviours observed in GW calves in the present study suggest that despite having an increased solid feed intake, they were not satiated throughout the period of milk step-down. This finding raises questions surrounding feeding level, duration, and age at gradual weaning of limit-fed calves. As indicated by Sweeney et al. [4], concentrate intake was increased in calves weaned gradually from 19 days of age, but calves were unable to compensate for the reduction in energy from milk, leading to an overall reduction in digestible energy intake and resulting in poor performance. It could be that if gradual weaning had commenced when calves were older, feeder behaviour differences between treatments may not have been as pronounced. The potential benefit of reducing latency in solid feed consumption and impact on post wean ADG could possibly be diminished by the increase in behaviours associated with hunger and their inherent impact on calf welfare. Additionally, as discussed by Sangha et al. [28], previous research has indicated that providing a consistent reinforcement schedule results in improved learning than a partial reinforcement schedule. As such, it could be that AW calves were better able to predict and learn when MR would be available and adjust their visits to the milk feeder accordingly, whereas GW calves had to adapt to a changing volume of MR between days 36 and 56.

Rosenberger et al. [10] compared effects of gradual weaning in calves offered four different levels of milk allowances ranging from 6–12 L/day. Gradually weaned calves in the present study exhibited similar pre-wean concentrate intakes to those provided with 6 L/milk per day, however in contrast to the previous study, time spent in and number of unrewarded visits to the milk feeder in the present study was comparable to those of calves fed 10 or 12 L milk/day. The disparity in automatic feeder behaviour displayed between the two studies could be due to group size, with seven calves per pen more in the present study than that described by Rosenberger et al. [10]. Borderas et al. [29] observed an increase in unrewarded milk feeder visits in limit-fed calves when compared with calves offered greater volumes of milk replacer. This lowers milk feeder efficiency, a finding echoed in the gradually weaned calves in the present study.

The sharp rise and fall in unrewarded visits to the milk feeder in AW calves following abrupt weaning in the present study was expected and comparable to the pattern in calves weaned abruptly from high and low milk allowances as described by Nielsen et al. [3]. In the present study, within 5 days AW calves had reduced the number of visits to the milk feeder to 17% of those observed on the day of complete milk withdrawal, this compared to 37% following complete weaning in GW calves. The rapid decrease in visits to the milk feeder in AW calves could suggest that although abrupt weaning does provoke an immediate, strong behavioural response, calves are able to recover more quickly than calves that have experienced repeated reductions in milk replacer allowance. However, again, the difference in calf age at weaning between the two treatment groups in the present study must be considered. As discussed by Miller-Cushon and DeVries [30], the impact of early management practices on feeding behaviour can have longer term implications for health, welfare and performance. Further examination of diurnal feeding patterns could help to develop optimum feeding strategies for calves of varying ages and under differing management regimes.

### 4.3. Physiological and Behavioural Measures

Van Reenen et al. [31] suggested that consistent individual differences exist between how calves respond to challenges, thus there is a need to examine the underlying physiological and behavioural responses resulting from commonly encountered stressors such as weaning. As indicated by Theurer et al. [7], the use of several monitoring techniques has the potential to provide a more accurate indicator of animal well-being. The combination of novel monitoring technologies as used in the present study provided the opportunity to measure behavioural and physiological responses to weaning method on an individual basis.

Previous research has identified heart rate variability (HRV) as a means of providing an indication of internal response to physiological, environmental and psychological stressors [32,33]. A decrease in root mean square of successive differences (RMSSD) as measured in a resting animal is reflective of vagal tone and considered indicative of increased stress [13,34]. In the present study, gradually weaned calves tended to exhibit reduced RMSSD values when compared to abruptly weaned calves following withdrawal of MR. This, and the accompanying automatic feeder behaviour during the period of milk step-down, could suggest that gradually weaned calves were experiencing an underlying level of stress as a result of feeding regime.

Automated recording of lying behaviour and activity has previously been demonstrated as a viable method of providing quantitative evidence of calf health and welfare [35]. Comparable to that found by Budzynska and Weary [36], abrupt withdrawal of milk replacer produced a strong and immediate response, with AW calves in the present study displaying a decrease in lying time following complete milk withdrawal. The decrease in lying time was likely as a result of the increase in visits to the milk feeder and occupation of the concentrate feeder. Gradually weaned calves did not display any significant increases in activity following withdrawal of milk. However, this is likely due to the fact that these calves were on low levels of milk replacer immediately prior to weaning and were already established on concentrate feed meaning there was no acute increase in occupation of the concentrate feeder. As with effects on feeder behaviour and HRV, the impact of weaning on AW calf activity was short lived, the effect generally limited to the time at which concentrate intakes increased to such a point that calves were satiated. It must be remembered, however, to reflect a commercially useable gradual weaning protocol and to ensure calves received the same total amount of milk powder, GW calves were completely weaned 1 week later than AW calves. As such, it is possible that physiological and behavioural results may have been influenced by calf age. Future research could potentially compare behavioural and physiological responses of calves offered the same total amount of milk powder and completely weaned at the same age using either gradual weaning by dilution or abrupt weaning.

## 5. Conclusions

Results suggest that calves fed according to the MR feeding level as described in this study can be weaned either gradually from 36 days of age with complete withdrawal of milk at 57 days of age or abruptly at 50 days of age without significant impact on live weight at 63 days of age. Performance differences throughout the experimental period were as expected and reflected calves’ ability to compensate for reduction or removal of milk replacer with consumption of concentrate. However, the information derived from a combination of non-invasive monitoring technologies has suggested gradual weaning of calves from 36 days of age may have experienced a more prolonged sense of frustration than that experienced by abruptly weaned calves. This study highlights the benefits of incorporating novel, non-invasive technologies to further elucidate effects of weaning method on dairy calf welfare, this potentially helping to inform best practice guidelines for weaning of calves fed moderate amounts of milk replacer. The study further highlights how age at weaning could play a role in both feeder behaviour and performance. Future work could consider using on-farm monitoring technologies to investigate the impact of pre-weaning MR feeding level, age at weaning and duration of weaning period in combination with other common management practices on calf well-being.

## Figures and Tables

**Table 1:** Colostrum/transition milk (TM) and milk replacer (MR) feeding plan for calves allocated to either a gradual or abrupt weaning protocol.

Age (d)	Weaning Method (L/Day)		No. of Feeds/d
	Gradual	Abrupt	
0–3	4 (colostrum/TM)	4 (colostrum/TM)	2
4 ^1^	4 (TM + MR)	4 (TM + MR)	2
5–8	4	4	Automatic feeder
9–35	6	6	Automatic feeder
36–49	6 reducing to 3.6	6	Automatic feeder
50–56	3.6 reducing to 2	-	Automatic feeder

^1^ Mix of half TM and half milk replacer supplied to calf.

**Table 2:** Chemical composition of milk replacer, concentrate and straw offered to calves allocated to a gradual or abrupt weaning protocol throughout the experimental period.

Parameter	Milk Replacer	Concentrate	Straw
Dry matter (g/kg Fresh)	963	965	948
Nitrogen (g/kg DM)	37.2	32.4	5.3
NDF (g/kg DM)	-	255.1	898.7
ADF (g/kg DM)	-	122.5	518.1
Ash (g/kg DM)	71.0	65.4	44.0
Ether extract (g/kg DM)	206.8	33.9	13.0
Gross energy (MJ/kg DM)	21.5	18.2	18.8

**Table 3:** Average daily and total intake of concentrate and milk replacer (on DM basis) of calves allocated to either a gradual or abrupt weaning protocol.

Parameter	Weaning Plan (WP)			*p*-Value		
	Gradual	Abrupt	SED	WP	Day	WP × Day
Daily Intake (gDM/day)						
Milk Replacer						
day 5–49	754	806	5.92	<0.001	<0.001	<0.001
Concentrate						
day 36–49	455	235	35.96	<0.001	<0.001	<0.001
day 50–56	1160	1557	84.46	<0.001	<0.001	<0.001
day 57–62	2280	2188	78.71	0.250	<0.001	0.715
Total Intake (kgDM)						
Milk Replacer	36.4	36.3	0.38	0.685	-	-
Concentrate						
day 5–49	9.1	4.1	0.79	<0.001	-	-
day 5–56	18.8	13.4	1.39	<0.001	-	-
day 5–62	32.6	26.5	1.79	0.001	-	-
day 36–49	6.6	3.1	0.25	<0.001	-	-
day 50–56	8.1	10.9	0.68	<0.001	-	-
day 57–62	13.7	13.1	0.49	0.266	-	-
Combined Dry matter intake (kgDM)						
day 5–56	55.2	49.6	1.47	<0.001	-	-
day 5–62	69.0	62.8	1.89	0.001	-	-

**Table 4:** Live weight, average daily gain, feed conversion efficiency and total feed costs of calves allocated to either a gradual or abrupt weaning protocol.

Parameter	Weaning Plan			*p*-Value
	Gradual	Abrupt	SED	
Live weight ^1^ (kg)				
day 50	70.1	69.2	1.16	0.354
day 57	75.9	74.5	1.12	0.215
day 63	83.4	81.2	1.28	0.085
ADG ^2^ (kg/day)				
day 7–35	0.59	0.57	0.023	0.393
day 7–63	0.68	0.65	0.019	0.079
day 36–49	0.66	0.71	0.036	0.163
day 50–56	0.90	0.39	0.078	<0.001
day 57–62	0.86	1.00	0.089	0.150
FCE ^3^ (kg gain/kg DMI)				
day 7–35	0.64	0.62	0.025	0.514
day 7–63	0.55	0.57	0.016	0.151
day 36–49	0.53	0.64	0.029	<0.001
day 50–56	0.47	0.21	0.126	0.049
day 57–62	0.32	0.42	0.043	0.031

^1^ Live weight (kg) as measured by manual weighbridge; ^2^ ADG = Average daily gain as calculated by regression using daily live weight from half body weight scales; ^3^ FCE = Feed conversion efficiency calculated as kg of BW gain/kg of total dry matter intake (DMI).

**Table 5:** Daily lying duration, number of lying bouts and lying bout duration over the pre-and post-weaning periods.

Parameter	Weaning Plan (WP)			*p*-Value		
	Gradual	Abrupt	SED	WP	Day (D)	WP × D
Daily total lying time (min/d)						
Pre-Abrupt (day 45–49)	1032	1037	14.91	0.871	0.984	0.713
Post-Abrupt (day 50–55)	1046	997	12.85	<0.001	0.012	0.105
Pre-Gradual (day 52–56)	1046	1025	16.62	0.193	0.123	0.154
Post-Gradual (day 57–62)	1037	1050	11.84	0.234	0.129	0.428
Number of daily lying episodes						
Pre-Abrupt (day 45–49)	16.16	17.45	0.890	0.188	0.867	0.579
Post-Abrupt (day 50–55)	17.17	15.03	0.708	0.004	0.016	0.095
Pre-Gradual (day 52–56)	17.24	15.69	0.715	0.066	0.032	0.053
Post-Gradual (day 57–62)	17.01	16.59	0.821	0.404	0.004	0.234
Lying bout duration (min)						
Pre-Abrupt (day 45–49)	66.56	61.98	3.813	0.276	0.416	0.897
Post-Abrupt (day 50–55)	64.24	68.94	2.741	0.100	0.034	0.160
Pre-Gradual (day 52–56)	61.15	71.59	2.944	0.001	0.053	0.639
Post-Gradual (day 57–62)	63.53	68.42	4.424	0.239	0.017	0.656

## Supplementary Materials

The following are available online at https://www.mdpi.com/2076-2615/9/11/895/s1, Figure S1: Daily concentrate intake (kg DM/day) of calves fed according to a gradual or abrupt weaning plan. 1 AW = day of complete withdrawal of milk replacer in abruptly weaned calves (d50). 2 GW = day of complete withdrawal of milk replacer in gradually weaned calves (d57). Figure S2: Live weight of calves weaned either abruptly or gradually as measured on a daily basis by automatic half bodyweight scale. Figure S3: Diurnal occupation of the milk (a) and concentrate (b) feeders (min/h) between days 36 and 62 in calves weaned either gradually between 36 and 57 days of age or abruptly at 50 days of age.

## References

[1] Weary D.M., Jasper J., Hötzel M.J. (2008). Understanding weaning distress. Appl. Anim. Behav. Sci..

[2] Hulbert L.E., Moisa S.J. (2016). Stress, immunity, and the management of calves. J. Dairy Sci..

[3] Nielsen P.P., Jensen M.B., Lidfors L. (2008). Milk allowance and weaning method affect the use of a computer controlled milk feeder and the development of cross-sucking in dairy calves. Appl. Anim. Behav. Sci..

[4] Sweeney B.C., Rushen J., Weary D.M., de Passille A.M. (2010). Duration of weaning, starter intake, and weight gain of dairy calves fed large amounts of milk. J. Dairy Sci..

[5] Overvest M.A., Crossley R.E., Miller-Cushon E., Devries T. (2018). Social housing influences the behavior and feed intake of dairy calves during weaning. J. Dairy Sci..

[6] Jasper J., Budzynska M., Weary D.M. (2008). Weaning distress in dairy calves: Acute behavioural responses by limit-fed calves. Appl. Anim. Behav. Sci..

[7] Theurer M.E., Amrine D.E., White B.J. (2013). Remote Noninvasive Assessment of Pain and Health Status in Cattle. Vet. Clin. N. Am.-Food Anim. Pract..

[8] Rushen J., Chapinal N., de Passille A.M. (2012). Automated monitoring of behavioural-based animal welfare indicators. Anim. Welf..

[9] de Passille A.M., Rushen J. (2012). Adjusting the weaning age of calves fed by automated feeders according to individual intakes of solid feed. J. Dairy Sci..

[10] Rosenberger K., Costa J.H.C., Neave H.W., von Keyserlingk M.A.G., Weary D.M. (2017). The effect of milk allowance on behavior and weight gains in dairy calves. J. Dairy Sci..

[11] De Paula Vieira A., Guesdon V., de Passillé A.M., von Keyserlingk M.A.G., Weary D.M. (2008). Behavioural indicators of hunger in dairy calves. Appl. Anim. Behav. Sci..

[12] Clapp J.B., Croarkin S., Dolphin C., Lyons S.K. (2014). Heart rate variability: A biomarker of dairy calf welfare. Anim. Prod. Sci..

[13] von Borell E., Langbein J., Despres G., Hansen S., Leterrier C., Marchant-Forde J., Marchant-Forde R., Minero M., Mohr E., Prunier A. (2007). Heart rate variability as a measure of autonomic regulation of cardiac activity for assessing stress and welfare in farm animals—A review. Physiol. Behav..

[14] Stewart M., Verkerk G.A., Stafford K.J., Schaefer A.L., Webster J.R. (2010). Noninvasive assessment of autonomic activity for evaluation of pain in calves, using surgical castration as a model. J. Dairy Sci..

[15] McEwan A.D., Fisher E.W., Selman I.E., Penhale W.J. (1970). A turbidity test for estimation of immune globulin levels in neonatal calf serum. Clin. Chim. Acta.

[16] Dunn A., Duffy C., Gordon A., Morrison S., Argűello A., Welsh M., Earley B. (2018). Comparison of single radial immunodiffusion and ELISA for the quantification of immunoglobulin G in bovine colostrum, milk and calf sera. J. Appl. Anim. Res..

[17] Dunn A., Ashfield A., Earley B., Welsh M., Gordon A., McGee M., Morrison S.J. (2017). Effect of concentrate supplementation during the dry period on colostrum quality and effect of colostrum feeding regimen on passive transfer of immunity, calf health, and performance. J. Dairy Sci..

[18] Quigley J.D., Wolfe T.A., Elsasser T.H. (2006). Effects of additional milk replacer feeding on calf health, growth, and selected blood metabolites in calves. J. Dairy Sci..

[19] McGuirk S.M., Peek S.F. (2014). Timely diagnosis of dairy calf respiratory disease using a standardized scoring system. Anim. Health Res. Rev..

[20] Finney G., Gordon A., Scoley G., Morrison S.J. (2018). Validating the IceRobotics IceQube tri-axial accelerometer for measuring daily lying duration in dairy calves. Livest. Sci..

[21] Task Force of the European Society of Cardiology (1996). Heart rate variability: Standards of measurement, physiological interpretation and clinical use. Task Force of the European Society of Cardiology and the North American Society of Pacing and Electrophysiology. Circulation.

[22] Kaufmann T., Sütterlin S., Schulz S.M., Vögele C. (2011). ARTiiFACT: A tool for heart rate artifact processing and heart rate variability analysis. Behav. Res. Methods.

[23] Khan M.A., Weary D.M., von Keyserlingk M.A. (2011). Invited review: Effects of milk ration on solid feed intake, weaning, and performance in dairy heifers. J. Dairy Sci..

[24] Steele M.A., Doelman J.H., Leal L.N., Soberon F., Carson M., Metcalf J.A. (2017). Abrupt weaning reduces postweaning growth and is associated with alterations in gastrointestinal markers of development in dairy calves fed an elevated plane of nutrition during the preweaning period. J. Dairy Sci..

[25] Baldwin R.L.V., McLeod K.R., Klotz J.L., Heitmann R.N. (2004). Rumen Development, Intestinal Growth and Hepatic Metabolism In The Pre- and Postweaning Ruminant. J. Dairy Sci..

[26] Nielsen B.L. (1999). On the interpretation of feeding behaviour measures and the use of feeding rate as an indicator of social constraint. Appl. Anim. Behav. Sci..

[27] Enriquez D., Hotzel M.J., Ungerfeld R. (2011). Minimising the stress of weaning of beef calves: A review. Acta Vet. Scand..

[28] Sangha S., McComb C., Scheibenstock A., Johannes C., Lukowiak K. (2002). The effects of continuous versus partial reinforcement schedules on associative learning, memory and extinction in Lymnaea stagnalis. J. Exp. Biol..

[29] Borderas T.F., de Passille A.M., Rushen J. (2009). Feeding behavior of calves fed small or large amounts of milk. J. Dairy Sci..

[30] Miller-Cushon E.K., DeVries T.J. (2015). Invited review: Development and expression of dairy calf feeding behaviour. Can. J. Anim. Sci..

[31] Van Reenen C.G., O’Connell N.E., Van der Werf J.T.N., Korte S.M., Hopster H., Jones R.B., Blokhuis H.J. (2005). Responses of calves to acute stress: Individual consistency and relations between behavioral and physiological measures. Physiol. Behav..

[32] Stewart M., Stafford K.J., Dowling S.K., Schaefer A.L., Webster J.R. (2008). Eye temperature and heart rate variability of calves disbudded with or without local anaesthetic. Physiol. Behav..

[33] Mohr E., Langbein J., Nurnberg G. (2002). Heart rate variability—A noninvasive approach to measure stress in calves and cows. Physiol. Behav..

[34] Kovacs L., Jurkovich V., Bakony M., Szenci O., Poti P., Tozser J. (2014). Welfare implication of measuring heart rate and heart rate variability in dairy cattle: Literature review and conclusions for future research. Animal.

[35] Swartz T.H., McGilliard M.L., Petersson-Wolfe C.S. (2016). Technical note: The use of an accelerometer for measuring step activity and lying behaviors in dairy calves. J. Dairy Sci..

[36] Budzynska M., Weary D.M. (2008). Weaning distress in dairy calves: Effects of alternative weaning procedures. Appl. Anim. Behav. Sci..

