# Supplementary material for: Performance and Behavioural Responses of Group Housed Dairy Calves to Two Different Weaning Methods
Source: Animals (Basel). 2019 Nov 1;9(11):895. doi: 10.3390/ani9110895 (PMC6912208; doi:10.3390/ani9110895)
Supplement: Supplementary file 1 [file animals-09-00895-s001.pdf]

Performance and behavioural responses of group housed dairy calves to two different weaning methods

Gillian Scoley [1,2*], Alan Gordon [3] and Steven Morrison [1]

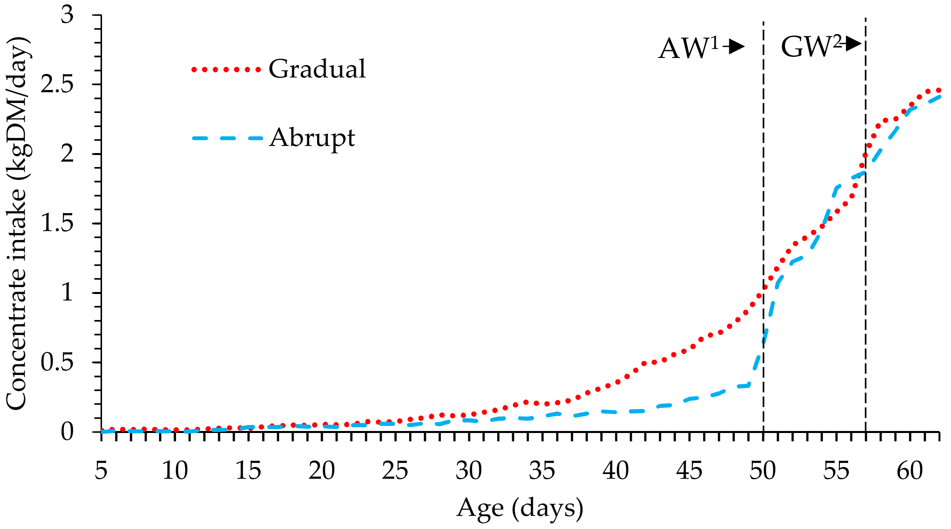

Figure S1. Daily concentrate intake (kg DM/day) of calves fed according to a gradual or abrupt weaning plan. [1] AW = day of complete withdrawal of milk replacer in abruptly weaned calves (d50). [2] GW = day of complete withdrawal of milk replacer in gradually weaned calves (d57).

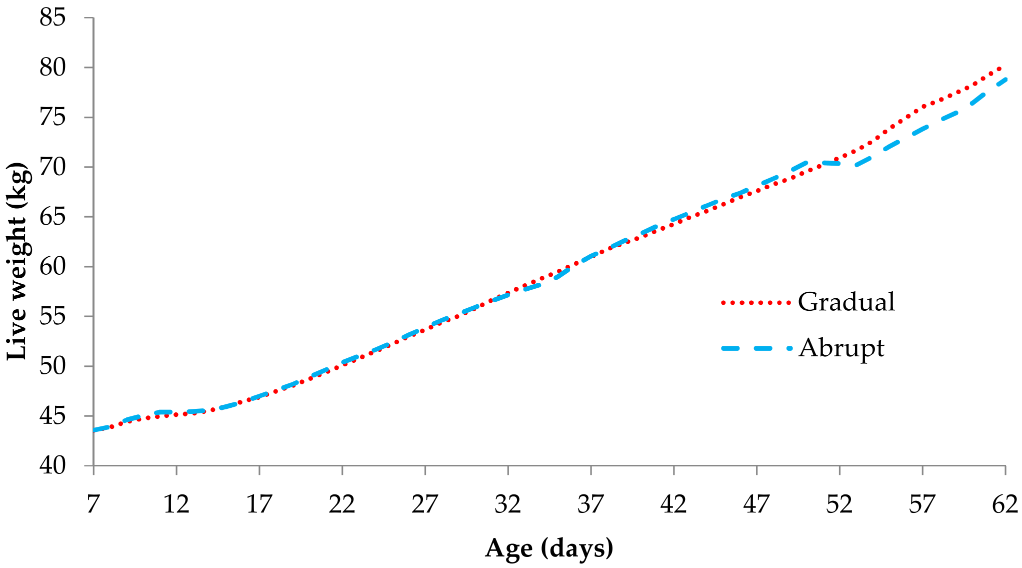

Figure S2. Live weight of calves weaned either abruptly or gradually as measured on a daily basis by automatic half bodyweight scale.

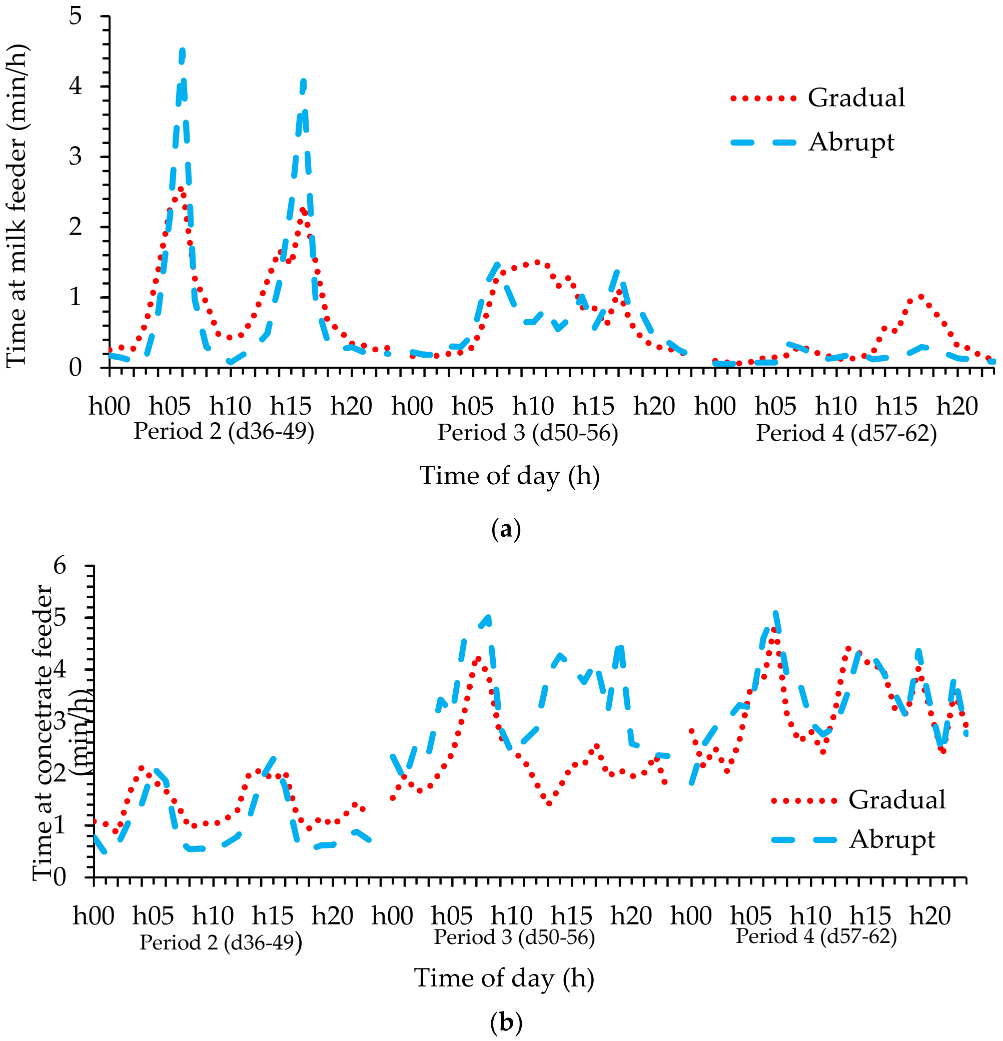

Figure S3. Diurnal occupation of the milk (**a**) and concentrate (**b**) feeders (min/h) between days 36 and 62 in calves weaned either gradually between 36 and 57 days of age or abruptly at 50 days of age.

Feeding behaviour and time of day

Gradually weaned calves spent increased time per hour in both the milk (Figure S3a; $p = 0.046$) and concentrate feeders (Figure S3b; $p < 0.001$) between days 36 and 49. Abruptly weaned calves displayed two peak times of increased milk feeder occupation when compared with gradually weaned calves, this being 6 a.m. and 3–4 p.m. ($p < 0.001$). An increase in duration of time spent in the milk feeder per hour was seen in GW calves between 03:00 and 04:00, 07:00 and 08:00, 10:00 and 14:00 and 17:00 (Figure S3a; $p < 0.001$). Although a similar pattern of occupation of the concentrate feeder during Period 2 (Figure S3b; day 36–49) was observed between treatments, GW calves consistently occupied it for a longer duration per hour than AW calves (Figure S1b; $p < 0.001$).

In Period 3s (day 50–56), similar to the pattern displayed in Period 2, AW calves spent longer in the milk feeder at 06:00, 17:00 and 19:00 compared with GW calves (Figure S3a; $p < 0.001$). GW calves' occupation of the milk feeder per hour was more prevalent than AW calves between 08:00 and 13:00 (Figure S3b; $p < 0.001$). During Period 3, AW calves spent an increased amount of time per hour in the concentrate feeder when compared with GW calves (Figure S3b; $p < 0.001$), this particularly apparent between 12:00 and 19:00 ($p < 0.001$). There was an effect of treatment in Period 4, with GW calves spending an increased time per hour in the milk feeder than AW calves (Figure S3a; $p < 0.001$). Increased occupation of the milk feeder by GW calves was observed on an hourly basis between 14:00 and 19:00 (Figure S3a; $p < 0.001$). There was no effect of treatment or treatment x hour of day on time spent in the concentrate feeder during Period 4.
